# Comparative randomized clinical trial on postoperative pain in circumcision with ultrasound-guided versus conventional anesthetic block

**DOI:** 10.1016/j.jped.2025.05.004

**Published:** 2025-06-19

**Authors:** Isabela P. Moraes, Flavia P. Payan, Camila G. Fachin, Rogério de Fraga

**Affiliations:** a Pediatric surgeon, Federal University of Parana (UFPR), Curitiba-PR, Brazil; b Medical student, Federal University of Parana (UFPR), Curitiba-PR, Brazil; c Urological surgeon, Federal University of Parana (UFPR), Curitiba-PR, Brazil

**Keywords:** Phimosis, Plastibell®, Circumcision, Postoperative pain, Ultrasound

## Abstract

**Objective:**

Circumcision is a common surgical procedure worldwide, with indications ranging from medical to cultural-religious contexts. Effective pain control is crucial to reduce analgesic use and improve patient safety. Recent advances include the use of the Plastibell® device and ultrasound-guided dorsal penile nerve block, aimed at minimizing surgical time and complications. This study compares postoperative pain in patients undergoing circumcision with either the landmark dorsal penile nerve block (blind block) or ultrasound-guided block.

**Methods:**

In this prospective, randomized study, patients aged 3–14 years undergoing elective circumcision were assigned to receive either anesthetic technique. Pain was assessed using physiological parameters (heart rate variation, movement during surgery) and subjective measures (Wong-Baker scale) at multiple time points, along with analgesic consumption. The sample was subdivided into patients aged ≤5 years and >5 years.

**Results:**

Pain scores before hospital discharge were higher in patients under 5 years. The blind block was faster to perform but had a higher incidence of hematomas and a trend toward greater block failure, indicated by increased heart rate, patient movement, and opioid use, although differences were not statistically significant. Ultrasound-guided blocks showed fewer complications and a tendency for better pain control.

**Conclusion:**

Both anesthetic techniques provide comparable pain control in circumcision; however, ultrasound guidance may reduce complications and improve block success, supporting its use as a safe and effective alternative to the conventional method.

## Introduction

Phimosis is characterized by the inability to retract the foreskin over the glans. It is considered physiological up to five years of age and is observed in 96 % of newborns. The natural process of foreskin retraction is due to the progressive dissociation of the preputial epithelium from the glans epithelium, a process influenced by factors such as inflammation, trauma, and infection.^1^^,^^2^ When phimosis persists beyond the age of five or is associated with symptoms, it is considered pathological.

The main complications of untreated phimosis include balanoposthitis, urinary tract infections, painful erections, and an increased risk of penile cancer in adulthood.^2^ The primary treatment for pathological phimosis is circumcision, a surgical procedure that involves removing the foreskin. Circumcision can be performed using various techniques, including the classic dissection method and the use of devices such as the Plastibell®.^3^

Pain management, especially in pediatric patients, is a crucial concern due to the delicate nature of the procedure and the patient’s age. Recent advances in anesthetic techniques, such as ultrasound-guided penile nerve blocks, have been developed to minimize pain and improve postoperative outcomes.^4^ However, there is still debate regarding the effectiveness of these techniques compared to traditional methods.

This study aims to evaluate the postoperative pain in patients undergoing circumcision, comparing the landmark dorsal penile nerve block (blind block) with the ultrasound-guided technique (US-guided), to determine if the newer method offers significant advantages in terms of pain management and overall patient comfort.

## Methods

This study strictly adhered to all ethical principles of the Research Ethics Committee and the Statute of the Child and Adolescent. Following the established guidelines and procedures, it was reviewed and approved under the CAAE process: 28,805,320.0.1001.0096 and is registered at Universal Trial Number (UTN) as U1111–1312–6236. The confidentiality of all participant information was ensured, and informed consent (from legal guardians) and assent (for those over 10 years) were obtained.

**Figure 1:**
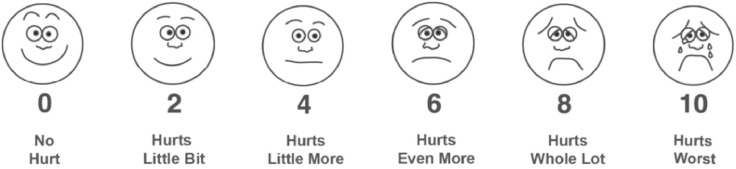
Original Wong-Baker Pain Assessment Scale.^4^

A prospective, randomized study was conducted with patients aged 3–14 years who had an indication for circumcision, from February 1, 2020, to July 1, 2024. No younger patients were included in this study, as the institutional protocol indicates the procedure only for those aged 3 years and above. Inclusion criteria were individuals who provided consent for participation and were able to complete the pain assessment using the Wong-Baker scale (Figure 1). Exclusion criteria were patients for whom the anesthetic approach was predetermined by the anesthesiologist.

Patients were divided into two groups, with random allocation to receive either the anatomic landmark (blind block) technique or the ultrasound-guided technique. The surgical techniques chosen for comparison were selected due to their reproducibility and frequent use in clinical practice and the literature worldwide. Patients were randomly assigned. After agreeing to participate, an envelope containing the assigned anesthetic technique was drawn. The content of the envelope was revealed only in the surgical center.

To minimize bias, a standard anesthetic protocol was defined in collaboration with the anesthesiology team. Inhalational anesthesia was induced and maintained throughout the surgery with sevoflurane until an adequate anesthetic plane was achieved, characterized by the absence of response to peripheral tactile stimulation, absence of the palpebral reflex, and capnographic stability.

The penile nerve block was performed using a solution composed of equal volumes of 1 % lidocaine and 0.25 % levobupivacaine without epinephrine, aiming to reduce latency and increase the duration of the anesthetic block. The infiltrated volume was calculated according to the patient's weight: 4 mg/kg for lidocaine and 2 mg/kg for levobupivacaine, with a maximum of 10 mL of the solution, regardless of weight, according to the experience of the anesthesiologists.

Randomization was conducted only after the anesthesiologist on duty approved the patient's participation in the study and after the patient and their legal guardians provided informed consent.

Blind blockages were performed by the anesthesiologist using anatomical landmarks, with a double puncture at the base of the penis and injection using a 22 G subcutaneous needle, used the same regardless of age, as it is the one routinely employed for blind blocks, administering half the volume on each side after the tactile sensation of passing through Scarpa's fascia, as described in the literature. Additionally, 1 mL was injected into the ventral aspect of the penis to block the frenulum.

**Figure 2:**
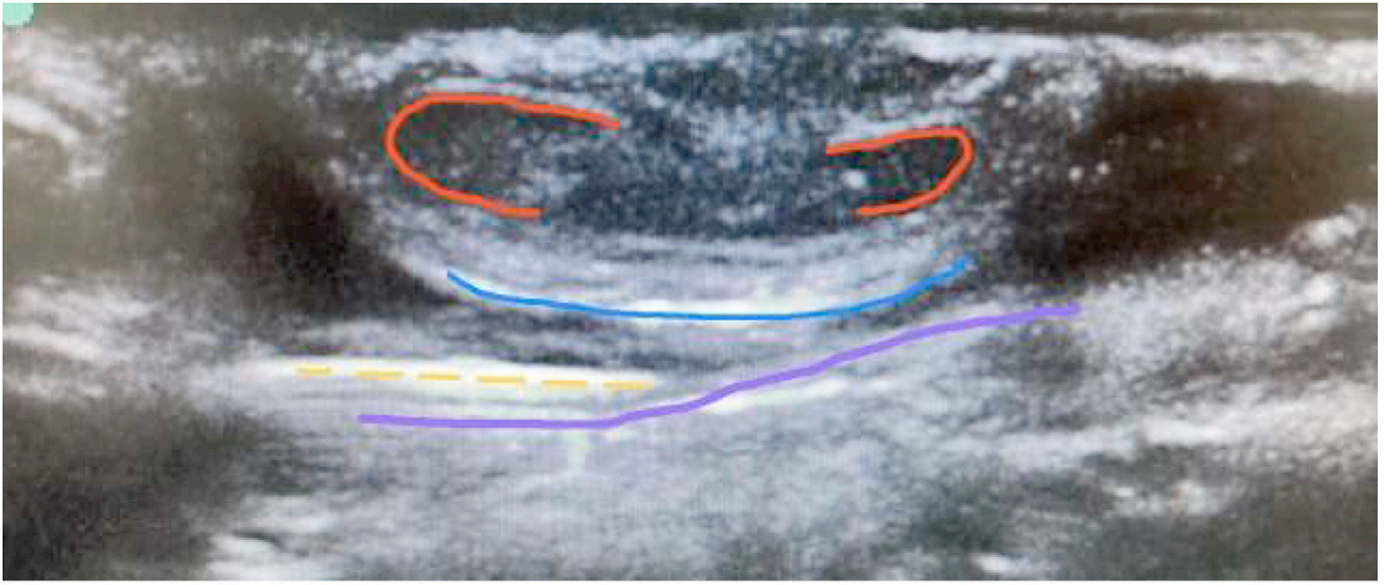
Needle positioning between the fasciae on the US-guided block. Highlighted are the corpora cavernosa in red, Buck's fascia in blue, and Scarpa's fascia in purple; the yellow dashed line indicates the needle positioned between these fasciae.

In US-guided blocks, a Logiq V2 VET-GE ultrasound with a 10 MHz linear probe and adjusted presets was used. After securing the penis with a micropore, asepsis and antisepsis were performed, followed by a single lateral puncture with in-plane visualization of the same 22 G needle until entry into the space between the fasciae, confirmed by visualization of the vessels. The same anesthetic solution was injected until the space between Buck's fascia and Scarpa's fascia was filled, a sign known as the ‘canoe sign’ (Figure 2).

The variables analyzed in this study were: the need for supplemental anesthetics during the surgical procedure, intraoperative time, hemodynamic parameters such as a 15 % increase in baseline heart rate during the procedure, patient movement during the surgical procedure (indicating emergence from the anesthetic plane), the need for analgesics in the post-anesthesia recovery room, pain scale assessment completed by the patient 1 hour after the procedure (corresponding to the half-life of the local anesthetic), and pain scale assessment at the first outpatient follow-up, 7 days after the procedure, to assess the late impact of potential complications.

In the present analysis, penile block failure was defined as an increase in heart rate above 15 % of baseline, movement during the procedure, or the need for opioid use during intraoperative or immediate postoperative periods. Additionally, the sample was subdivided into two age groups: one up to 5 years old and the other over 5 years old, considering cognitive-emotional developmental differences for self-reported pain.

### Statistical analysis

To descriptively explore the behavior of the present data, the authors used mean (±SD) and median (min-max) values for quantitative variables of interest. For qualitative variables, the authors expressed the behavior using absolute values and percentage of the total (%). For qualitative variables, the authors used the chi-square association test, applying Fisher's correction, when necessary, especially for cells with zero values. For all tests, p-values < 0.05 were considered sufficient to reject the null hypothesis and consider the result statistically significant. All statistical analyses, as well as the creation of graphs and tables, were performed using the statistical software JAMOVI, version 2.5.0, which is based on the R language.

## Results

A total of 60 patients underwent circumcision during the study period. One patient was excluded due to the use of opioids during anesthetic induction (Table 1). Of the included patients, 29 underwent a US-guided block, and 30 underwent a blind block. However, during follow-up, only 34 patients attended all outpatient consultations. This did not impact the execution of the study, as the collected variables were immediate and related to the hospital stay. Nevertheless, it highlights a deficiency in outpatient follow-up for pediatric patients, suggesting the need for strategies to ensure long-term patient engagement and monitoring.

**Table 1:** Epidemiological profile of the sample.

	US-guided (*N* = 29)	Blind blockage (*N* = 30)	p-value
Age			
Mean (SD)	6.86 (2.44)	6.38 (2.41)	0.452^⁎⁎^
Median [Min, Max]	6.00 [3.00, 13.0]	6.00 [3.00, 11.0]	
Surgical technique			
Conventional	10 (34.5 %)	15 (50.0 %)	0.346^⁎⁎⁎^
Plastibell	19 (65.5 %)	15 (50.0 %)	

The mean age of the patients was 6 years, ranging from 3 to 13 years. Only 8 patients required opioids postoperatively after pain assessment, with pain scores ranging from 1 to 6 one hour after the procedure, and a mean score of 1.29 (Table 2).

**Table 2:** Results and statistical analysis.

	US-guided (*N* = 29)	Blind blockage (*N* = 30)	p-value
Heart rate increase			
No	27 (93.1 %)	22 (73.3 %)	0.0937⁎⁎⁎
Yes	2 (6.9 %)	8 (26.7 %)	
Mobilization			
No	27 (93.1 %)	22 (73.3 %)	0.0937⁎⁎⁎
Yes	2 (6.9 %)	8 (26.7 %)	
Opioid use			
No	27 (93.1 %)	24 (80.0 %)	0.276⁎⁎⁎
Yes	2 (6.9 %)	6 (20.0 %)	
Pain 1 hour post-procedure			
Mean (SD)	2.48 (1.66)	2.87 (1.53)	0.359⁎⁎
Median [Min, Max]	2.00 [1.00, 6.00]	3.00 [1.00, 6.00]	
Pain before discharge			
Mean (SD)	2.00 (1.13)	2.60 (1.59)	0.1⁎⁎
Median [Min, Max]	2.00 [1.00, 5.00]	2.00 [0, 6.00]	
Pain at follow-up (7 days)			
Mean (SD)	1.64 (0.989)	1.83 (1.00)	0.487⁎⁎
Median [Min, Max]	1.00 [1.00, 4.00]	2.00 [1.00, 4.00]	
Time to surgery start			
Mean (SD)	13.7 (3.61)	11.8 (3.38)	0.0509*
Median [Min, Max]	14.0 [7.00, 20.0]	12.0 [7.00, 23.0]	
Surgery duration			
Mean (SD)	18.5 (7.25)	16.5 (5.91)	0.274*
Median [Min, Max]	17.5 [9.00, 35.0]	17.0 [7.00, 27.0]	
Age category			
≥ 5	20 (69.0 %)	16 (53.3 %)	0.335⁎⁎⁎
< 5	9 (31.0 %)	14 (46.7 %)	
Post-anesthesia hematoma			
No	29 (100 %)	26 (86.7 %)	0.129⁎⁎⁎
Yes	0 (0 %)	4 (13.3 %)	

⁎ T-test.

⁎⁎ Mann-whitney U.

⁎⁎⁎ Qui-square.

**Figure 3:**
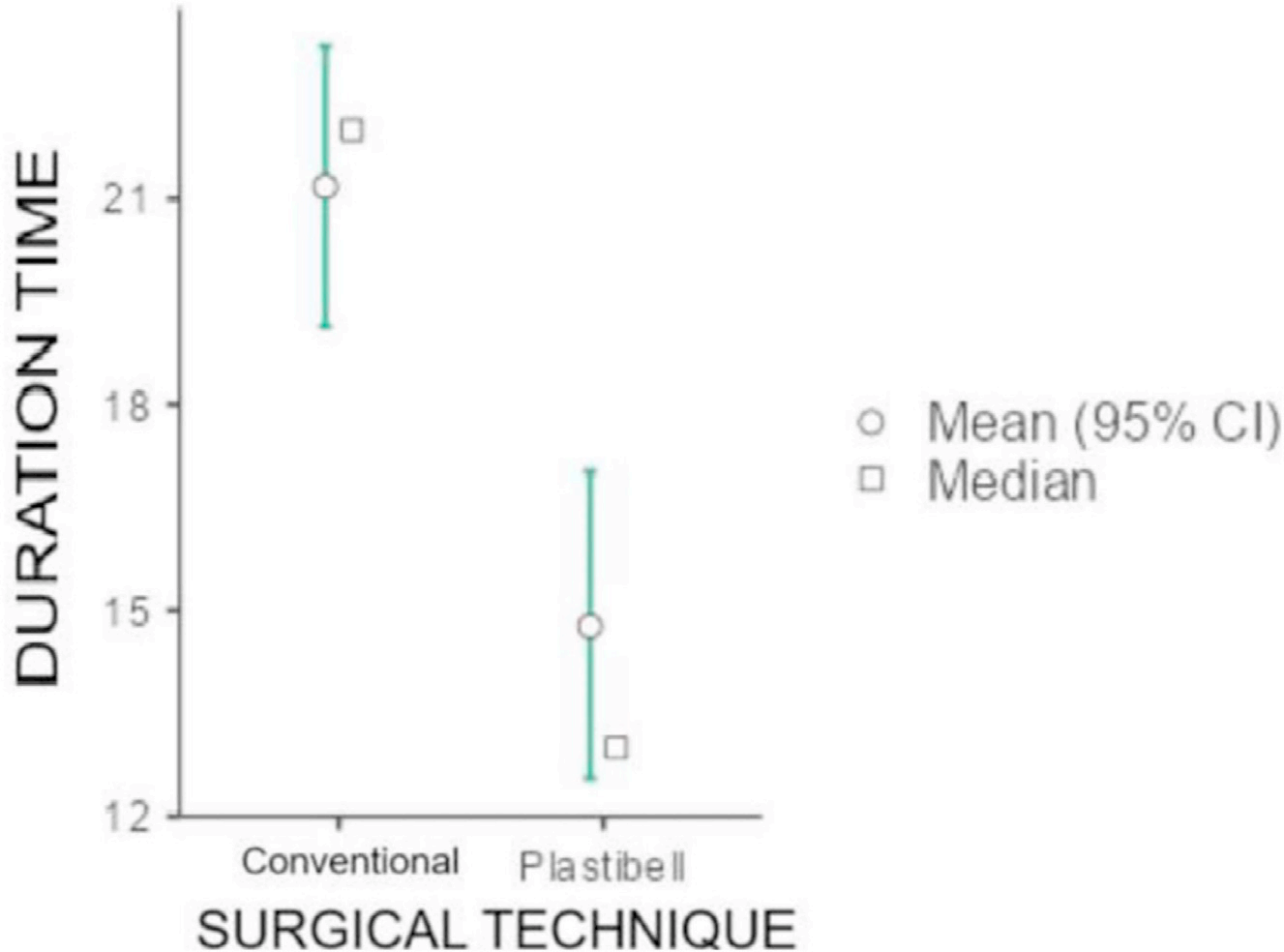
Duration of the surgical procedure. The scale duration time is minutes and represents the total duration of the procedure.

Regarding the surgical technique, the only statistically significant difference (*p* < 0.001) was in the duration of the procedure, with the conventional technique (open surgery) having an average duration of 21.2 min and the Plastibell technique 14.8 min (Figure 3).

Within the parameters that defined block failure (increase in heart rate above 15 % of baseline, movement during the procedure, and need for opioid use during intraoperative or immediate postoperative periods), no statistically significant result was found, but in all parameters, there was a higher incidence of failure within the blind block group.

**Figure 4:**
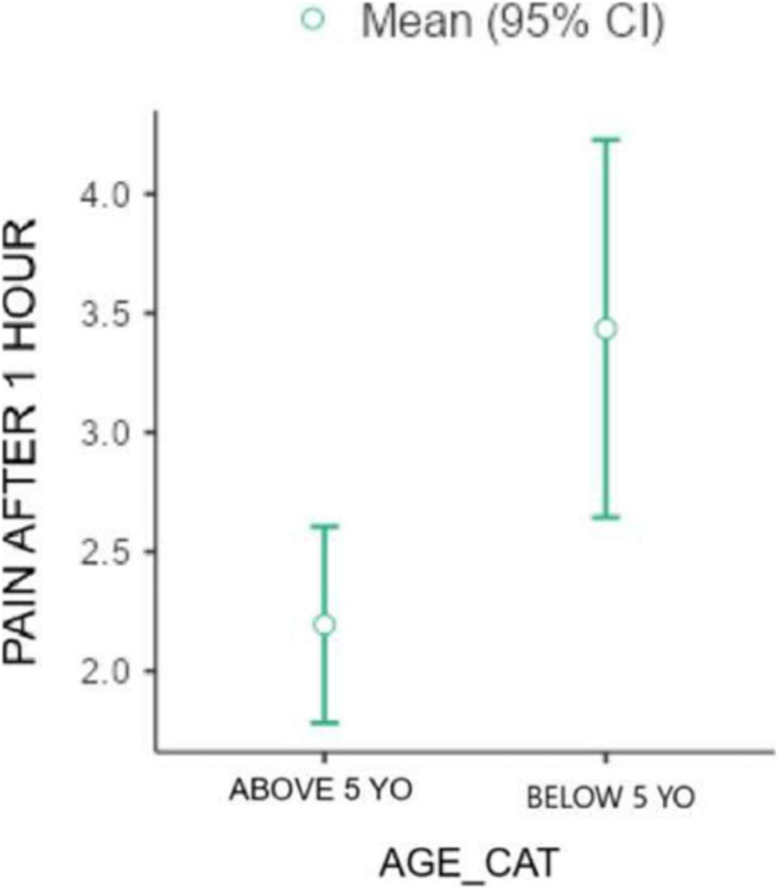
Pain after one-hour x age category. “Pain” is referred to as the score the patient indicated on the Wong-Baker scale.

Additionally, in all applications of the Wong-Baker scale to assess patients' pain after 1 hour (Figure 4), immediately before discharge, and at follow-up (7 days after the procedure) the mean value was higher in the blind block group compared to the US-guided block group (respectively 2.87/2.48, 2.6/2.0, 1.83/1.64), although these differences were not statistically significant.

When the authors analyzed the time to start the procedure, which included the time to perform the penile block, the mean time was 13.7 min in the US-guided group versus 11.8 min in the blind block group. Although not statistically significant, the p-value was 0.0509. However, when analyzing the total duration of the procedure, the p-value was higher, at 0.274, although the mean duration was also higher in the US group (18.5 vs. 16.5 min).

No complications occurred in the US-guided block group. However, in the blind block group, four patients (13.3 %) developed hematomas. Neither group experienced inadvertent intravenous anesthetic injection or local anesthetic toxicity.

## Discussion

The first dorsal penile nerve block was performed in 1972 by Bateman et al.^5^ Since then, this technique has been successfully used for anesthesia in penile surgeries, with no significant changes in its execution. However, the complication rate varies between 5 % and 15 %, with the most common being hematoma and local anesthetic toxicity.^6^

It was not until 2007 that Sandemann et al. proposed the possibility of performing this block under ultrasound guidance.^7^ Since then, various techniques have emerged, and some studies have sought to demonstrate their effectiveness. In Sandemann's article, 70 cases of US-guided block were reported without any failures. However, there is no prospective, randomized study in the literature directly comparing the two techniques.

In 2016, in an attempt to demonstrate the greater effectiveness of the US-guided method compared to the conventional method, Suleman et al.^8^ conducted a retrospective study with 16 patients who underwent each type of block. Although the data did not reach statistical significance, there was a trend toward a greater need for supplemental analgesia (1.8 times) and an increased risk of vascular complications (2 times) in the group subjected to the classic block. This trend was also observed and confirmed in the present study, with hematomas occurring only in the group subjected to the block without ultrasound guidance.

When evaluating the parameters of anesthetic block failure, the blind block group showed an increase in heart rate at the most painful stimulus of the procedure in 26.7 % of cases, compared to 6.9 % in the group subjected to the US-guided block, resulting in a 3.8 times higher likelihood of failure in the blind block group. Although this difference did not reach statistical significance, it suggests a potential clinical benefit of the ultrasound-guided technique.

The need for opioid use during the procedure or in the immediate postoperative period was also higher in the blind block group (16.7 %) compared to the US-guided group (6.9 %), reinforcing the trend observed in previous studies. The incidence of patient movement during the procedure, which may indicate inadequate anesthesia, was also higher in the blind block group (23.3 % versus 10.3 %). These findings, although not statistically significant, are clinically relevant and support the use of ultrasound guidance to increase the accuracy and effectiveness of the block. When used correctly, ultrasound allows visualization of neurovascular structures, providing safer anesthesia by reducing the risk of inadvertent vascular injection and vessel puncture, which can cause hematoma. It also increases the duration of the block by allowing anesthetic injection closer to the neural bundle.^9^

The neurovascular structures of the penis lie immediately beneath the deep fascia of the penis (Buck's fascia) on either side of the midline. To verify ultrasonographic findings and anatomy, Zadrazil et al., in 2023,^10^ dissected the penile region of three fresh cadavers, revealing a complex anatomy, particularly regarding the first branches of the pudendal nerve, which are responsible for innervating the penile frenulum and scrotal region. These anatomical variations were attributed to the 27 % failure rate of US-guided blocks in their study. A similar result was found in the present study, with a failure rate of 16.9 %.

A common concern with anesthetic blocks is the time required for their execution. Regarding the duration of the procedure, the time required to perform the ultrasound-guided block was slightly longer than for the blind block (13.7 min versus 11.8 min), with a p-value of 0.0509. However, when considering the total duration of the procedure, including surgical time, the difference was not significant (18.5 min for the US-guided group versus 16.5 min for the blind block group, *p* = 0.274). This suggests that the additional time required for ultrasound guidance does not significantly impact the overall duration of the procedure.

With adequate training, the time required for performing the anesthetic block decreased, suggesting that practice may lead to equalization of the time between the two types of blocks. However, further studies are needed to confirm this trend. The time between the completion of the penile blockage and the beginning of the procedure was established in collaboration with the anesthesiology team and adhered to in accordance with the anesthetic’s half-life of 5 min.

**Figure. 5:**
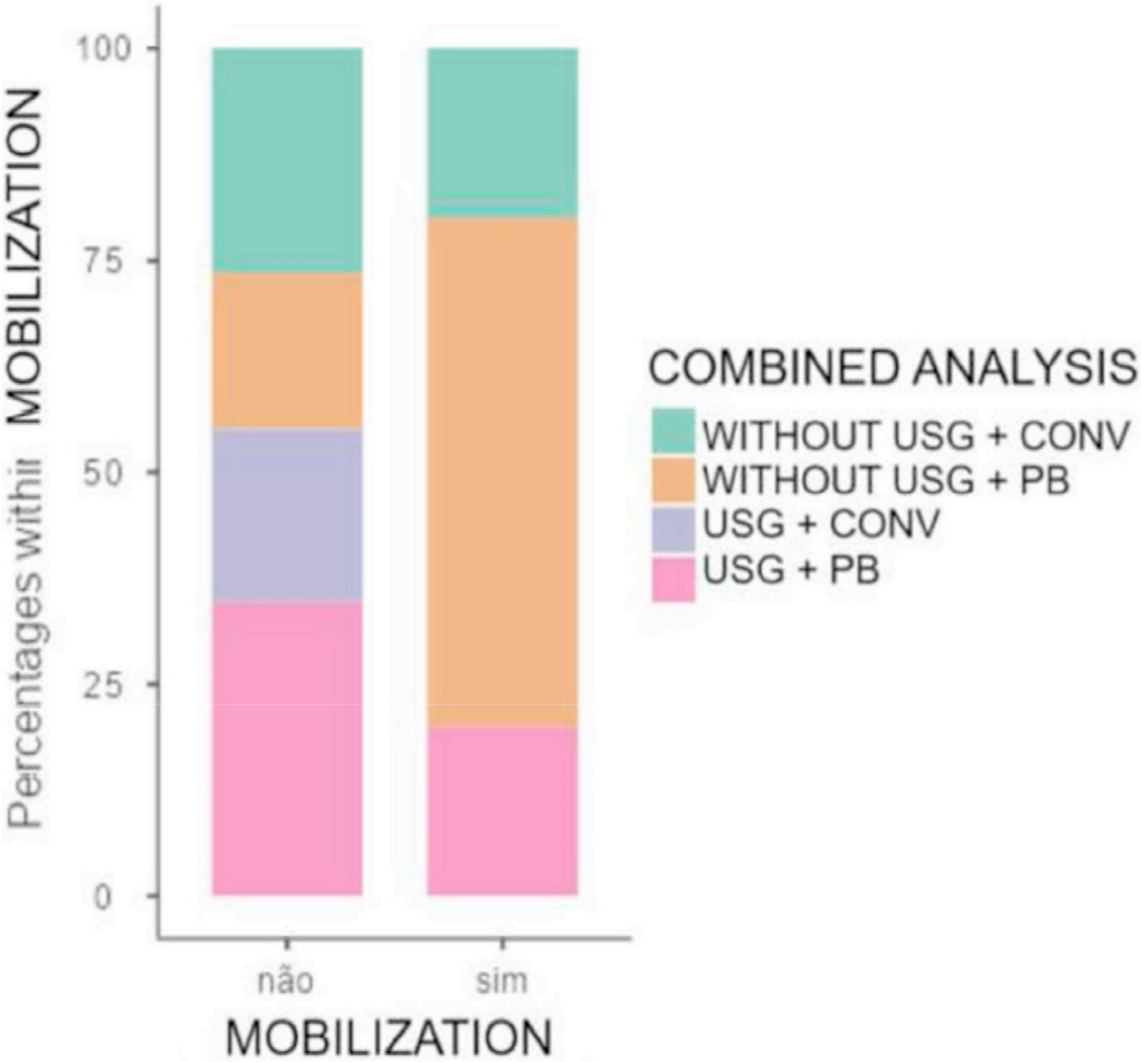
Comparison of mobilization between the groups.

The main factor with statistical significance studied was intraoperative mobilization, one of the criteria for anesthetic block failure. Mobilization was lower in the group with a US-guided block (Figure 5), likely because the injection was administered directly adjacent to the nerve, reducing the time needed for the blockade of ascending pain fibers to take effect.

Pain assessment using the Wong-Baker scale showed higher mean scores in the blind block group at all time points (1 hour after the procedure, before discharge, and at the 7-day follow-up), although these differences were not statistically significant. This may be related to the greater precision of the ultrasound-guided block, which allows for more accurate deposition of the anesthetic solution and potentially better pain control.

It is important to note that pain assessment in pediatric patients is challenging, especially in those under 5 years of age. The use of age-appropriate pain scales, such as the FLACC scale, is recommended for younger children who may have difficulty understanding or using self-report scales like the Wong-Baker scale.^4^^,^^11^ In the present study, all patients were able to complete the Wong-Baker scale, but future studies should consider the use of alternative scales for younger patients.

The formulation of an anesthesia protocol with the responsible physicians helped reduce potential biases. However, since this study was conducted in a university hospital with high resident turnover, efforts were made to minimize bias by ensuring the presence of a trained team member in all procedures to assist and address any questions.

The present study has some limitations, including the relatively small sample size and the loss of follow-up in some patients. Additionally, the subjective nature of pain assessment and the potential for observer bias should be considered when interpreting the results. Despite these limitations, the present findings contribute to the growing body of evidence supporting the use of ultrasound-guided penile nerve blocks as a safe and effective technique for circumcision in pediatric patients.

In conclusion, the ultrasound-guided penile nerve block demonstrated a trend toward better outcomes in terms of pain control and complication rates compared to the conventional blind technique, although these differences did not reach statistical significance. The additional time required for ultrasound guidance is minimal and does not impact the overall procedure duration. The authors recommend the use of ultrasound guidance for penile nerve block in circumcision, particularly in settings where resources and expertise are available.

During the preparation of this work, the author(s) used the Perplexity platform in order to improve language and readability. After using this tool/service, the author(s) reviewed and edited the content as needed and take(s) full responsibility for the content of the publication.

## Conflicts of interest

The authors declare no conflicts of interest.
